# Mental health training for correctional officers: a systematic review

**DOI:** 10.1192/bjo.2021.104

**Published:** 2021-06-18

**Authors:** Shaheen Darani, Sandy Simpson, Robert McMaster, Elena Wolff, Sarah Bonato, Graham Glancy, Jason Quinn

**Affiliations:** 1 University of Toronto and Centre for Addiction and Mental Health; 2 University of Toronto, MD Program; 3 Centre for Addiction and Mental Health; 4 Department of Psychiatry, University of Western Ontario, London

## Abstract

**Aims:**

Mental illness amongst prison inmates is a prevailing issue across the world, as mental illnesses are overrepresented in correctional facilities when compared to community populations. Despite this, correctional officers receive little to no training on how to respond to inmates with mental illness. Implementing mental health training could improve officer knowledge, skills, and attitudes toward inmates with mental illness. This could lead to improvements in risk management, humane treatment of inmates, and interprofessional collaboration with healthcare providers. There is limited research on the educational value of inmate mental health training programs for correctional officers. As far as we are aware, there have been no prior reviews of this literature. The goal of the present study is to review this literature to explore the nature and effectiveness of correctional officer mental health training programs.

**Method:**

Medical and criminal justice databases were searched for scientific articles describing correctional officer mental health training programs. All studies that included a measurable outcome on either correctional officer knowledge or inmate mental health were included in a final analysis. The review adhered to PRISMA guidelines for systematic reviews.

**Result:**

Of 1492 articles identified using search terms, 11 were included in the analysis. 6 articles described mental health education programs, 2 articles described skill-specific programs, and 3 articles described suicide prevention programs. Training programs reviewed content about mental illness, practical skills, and included didactic and experiential teaching modalities. The programs led to improvements in knowledge, skills, and attitudes amongst officers. Prior mental health attitudes, knowledge, and work experience did not correlate with improvements following training. Officers were more receptive to program facilitators with correctional or lived mental health experience. Experiential teaching was preferred to didactic teaching. A decline in training improvements occurred several months after training.

**Conclusion:**

There is limited but positive literature suggesting that structured training programs, particularly involving persons with lived experience and experiential components are beneficial. The decline in training improvements suggests need for ongoing education and systems change within correctional institutions to ensure sustainability of gains. In terms of limitations of this review, it is possible articles pertaining to correctional officer mental health training were not available on the databases searched or some programs may not be published. Studies were also limited in their outcome measurement, with no consistent tools, and no control groups. This review can guide the development, delivery, and contribute toward best practice guidelines for future inmate mental health training programs and studies.

